# Factors Affecting Acceptance of Participating in Clinical Trials of New Drugs: A National Cross‐Sectional Study

**DOI:** 10.1111/jebm.70176

**Published:** 2026-08-26

**Authors:** Shujie Dong, Mengmeng Liu, Yuanyuan Zhang, Jinzi Zhang, Qin Han, Xiaohan Chen, Yibo Wu, Wai‐kit Ming

**Affiliations:** ^1^ Department of Pharmacy Peking University Third Hospital Beijing China; ^2^ Department of Infectious Diseases and Public Health Jockey Club College of Veterinary Medicine and Life Sciences, City University of Hong Kong Hong Kong China; ^3^ Department of Surgery Faculty of Medicine The Chinese University of Hong Kong Hong Kong China; ^4^ Graduate School Harbin Medical University Harbin China; ^5^ School of Health Policy and Management Chinese Academy of Medical Sciences & Peking Union Medical College Beijing China; ^6^ Department of Nursing the Fourth Affiliated Hospital of School of Medicine and International School of Medicine, International Institutes of Medicine, Zhejiang University Yiwu China

**Keywords:** clinical trial, cross‐sectional study, new drug, public willingness

## Abstract

**Objective:**

Clinical trials are regarded as an essential method for developing new treatments and giving patients access to potentially effective medications that are still being studied. This study aimed to evaluate acceptance of participating in clinical trials of new drugs and to further explore the associated factors.

**Methods:**

A population‐based cross‐sectional study was conducted in China from June 20 to August 31, 2022. We collected demographic data and used visual analog scale scores to assess the public's willingness to accept clinical trials of new drugs (range 0–100). Linear regression analysis was adopted to evaluate the factors related to attitudes toward clinical trials of new drugs.

**Results:**

This survey contained 19,710 valid questionnaires to investigate acceptance of participating in clinical trials of new drugs. Participants showed low willingness to accept clinical trials of new drugs, with a mean score of 44.69 out of 100. The results showed that rural residence (*β* = 3.00), western China (*β* = 3.15), being married (*β* = 2.93), older age (31–59 years [*β* = 3.84], ≥60 years [*β* = 7.71]), having cancer or rare diseases (*β* = 5.15), higher monthly household income (>6000 yuan [*β* = 1.76]), medical‐related occupations (*β* = 8.01), anxiety status (moderate anxiety [*β* = 1.83]), moderate to severe anxiety [*β* = 2.66]), perceived stress (*β* = 0.51), health literacy (*β* = 0.21), self‐efficacy (*β* = 0.34), and media use (*β* = 0.42) were associated with higher acceptance of participating in clinical trials of new drugs. However, central China (*β* = –1.65), female sex (*β* = –2.29), chronic diseases (*β* = –2.45), and medical insurance (*β* = –3.78) were associated with lower acceptance of participating in clinical trials of new drugs. Subgroup analysis demonstrated consistent trends across age and chronic disease subgroups.

**Conclusions:**

This reveales low public willingness to accept clinical trials of new drugs. The willingness is affected by marital status, occupation, area of residence, income, health insurance, physical and mental health status, media use, and health literacy.

## 1 Introduction

The number of new drug approvals has had a general upward trend in recent years. Food and Drug Administration (FDA) approved a total of 247 drugs including small and macro‐molecules from 2018 to 2022 in United States [1]. According to the National Medical Products Administration (NMPA), China has approved a total of 136 innovative drugs from 2018 to 2023. Clinical trials of new drugs are active around the world. Clinical trials are regarded as a crucial approach to developing novel therapies and grant patients an opportunity to utilize potentially efficacious medicines that are still undergoing examination [2]. Through rigorous trial design and data analysis, they can assess the efficacy and safety of new drugs, provide better treatment options for patients, and give patients access to new drug interventions as well. It is reported that 2357 companies started clinical trials worldwide in 2023, rising from 993 in 2008. Among these, the proportion of trial launches by Chinese companies has increased significantly from 1% in 2008 to 28% in 2023, which demonstrates China's significant position in the global pharmaceutical research and development field [3].

Participation in clinical trials is crucial to their success as well as to benefits for participating communities [4]. Though clinical trials have significant benefits for patients, the acceptance of participating in clinical trials was reported to be low. 35 trials conducted in the United States from 2000–2020 revealed that 45% of patients declined to participate in the trials, in which alternative treatment choices or lack of interest were regarded as the primary reasons [5]. Similarly, only 44.6% of adults showed interest and accepted participation in COVID‐19 clinical trials [6]. The most frequently reported motives for clinical trial participation were “wanting to get better” and the standard of care not covered by medical insurance [5].

Previous studies have demonstrated that multiple factors such as age [6, 7, 8, 9, 10, 11, 12, 13, 14], gender [6, 8, 10, 11, 12], marital status [6, 14, 15], occupational status [6, 11], economic status [6, 8, 9, 11, 12, 16], health insurance [11], and health status [11, 13, 17] significantly influence people's acceptance of clinical trials of new drugs. Domestic studies have also examined related topics, including public awareness of and willingness to participate in drug clinical trials in inland cities of northern China [18] and attitudes and concerns among healthy volunteers participating in clinical trials in a city in central China [19]. However, many studies tend to be condition‐specific (e.g., cancer patients), therapy‐specific (e.g., COVID‐19 vaccines), or geographically limited, and may underrepresent the real‐world general population that clinical trials may involve. Evidence for the public's willingness regarding clinical trials of new drugs across multiple provinces in China remains limited.

Our study aimed to evaluate the public's willingness to participate in clinical trials of new drugs and to explore associated factors, using a population‐based cross‐sectional database in China.

## 2 Methods

### 2.1 Data Collection

This population‐based cross‐sectional study was conducted in China from June 20, 2022, to August 31, 2022. It included 148 cities, 202 districts or counties, 390 townships or streets, and 780 communities or villages in 23 provinces of China, with 19,710 questionnaires included in the final statistical analysis. The study used stratified sampling at the provincial, autonomous region, or municipality level and non‐proportional quota sampling at the community, village, or individual level to select a nationally representative sample of people in the general population.

Specific quota sampling criteria (every 100 samples) include: (1) age criteria: under 18 years: 8 ± 5 samples; between 19 and 24 years: 12 ± 5; between 25 and 30 years: 12 ± 5; between 31 and 40 years: 16 ± 5; between 41 and 50 years: 18 ± 5; between 51 and 60 years: 18 ± 5; between 61 and 70 years: 10 ± 5; aged 71 years and above: 6 ± 5; (2) gender criteria: the proportion between male and female is close to 1:1; (3) urban and rural distribution criteria: the proportion between urban samples and rural samples is close to 3:2. For this study respondents, the inclusion criteria were (1) aged ≥18 years; (2) having Chinese nationality; (3) a permanent resident of China (annual time away from country ≤1 month); (4) participants could complete the questionnaire on their own or with the help of an investigator; (5) participants were able to understand the meaning of the questionnaire as expressed by each entry; (6) voluntarily agreed to participate in the study and signed an informed consent form.

The exclusion criteria for respondents were (1) those who were confused or mentally abnormal; (2) those who had cognitive dysfunction (mental illnesses such as cognitive dysfunction was obtained through records in the community health Centre system and self‐reported by the respondents); (3) those involved in other similar research projects.

For each city, at least one trained team was assigned to collect information about behavioral factors, mental health status, personal health status, medical history, and related variables. The survey was conducted anonymously by distributing an electronic questionnaire designed on the Wenjuanxing platform in face‐to‐face interviews with the subjects. When face‐to‐face interviews were not suitable for personal reasons (e.g., respondents were quarantined at home due to COVID‐19 or were unable to complete the questionnaires independently for medical reasons), the investigators used online communication platforms to conduct video‐call surveys. Additionally, to ensure the quality and quantity of the face‐to‐face questionnaires, the survey team was composed of 23 survey coordinators and 660 survey members. Each survey coordinator was in charge of 23–30 survey members, and each survey member was responsible for 20–25 questionnaires. Ultimately, a total of 31,449 questionnaires were recovered, and the overall recovery rate was 80.6%. After adjustment for quota sampling based on Chinese demographic characteristics, 21,916 questionnaires met the criteria. Then we excluded 2710 persons under 18 years and 36 persons not in China in the past 3 months. Finally, our analysis included 19,710 questionnaires in statistical analysis.

This study encompasses all types of clinical trials, including but not limited to phase I, phase II, phase III, and phase IV trials. The objective of this study was to obtain a comprehensive understanding of the acceptability of participation across the full range of clinical trials. This study followed the Strengthening the Reporting of Observational Studies in Epidemiology (STROBE) reporting guidelines, and the study protocol was approved by the Ethical Review Committee of Shaanxi Health Culture Research Center (JKWH‐2022‐02; 2022‐K050). The questionnaire used in this study went through 38 expert consultations and 3 pre‐surveys to ensure the validity of the results. All participants provided written informed consent after receiving a thorough explanation of the research procedures.

### 2.2 Research Instrument

We used visual analog scale scores to assess the public's willingness to participate in clinical trials of new drugs (range 0–100). Higher scores indicate a higher willingness to participate in clinical trials of new drugs. The factors associated with people's acceptance of participating in clinical trials of new drugs are multifaceted. Among the potentially relevant factors were (1) basic personal information (gender, age, region, occupation, and education); (2) family status (marital status); (3) economic status (monthly household income, household debt, and insurance coverage); (4) health literacy (12‐item version health‐literacy scale and media use scale); and (5) physical and mental health status (chronic diseases [hypertension, diabetes, hyperlipidemia, etc.], anxiety scale, depression scale, perceived stress scale, perceived social support score, and European Quality of Life 5‐Dimension 5‐Level version score).

The 12‐item short‐form health literacy scale (HLS‐SF12) assesses a participant's ability to discover, understand, evaluate, and apply health‐related information to measure the participant's health literacy status. Items are rated from 1 to 4 (Likert‐type), where 1 means very difficult and 4 means very easy. In this study, the health literacy index score was standardized to 0–50 points, with higher scores representing higher health literacy [20, 21].

The Perceived Stress Scale‐4 (PSS‐4) measured overall levels of perceived stress. Each item is rated from 0 to 4, and total scores range from 0 to 16, with higher scores indicating higher perceived stress [22, 23].

The perceived social support score (PSSS score) was derived from three items adapted from the Perceived Social Support Scale, covering perceived support from family, friends, and significant others [24]. In the present questionnaire, each item was scored from 1 to 7, and total scores ranged from 3 to 21, with higher scores indicating greater perceived social support. This variable was included because social support may influence health‐related decision‐making and willingness to participate in clinical research.

The New General Self‐Efficacy Scale (NGSES) was used to measure the level of self‐efficacy. Each option in the Likert scale ranges from 1 to 5, with 1 indicating strongly disagree and 5 indicating strong agreement. Total scores range from 3 to 15, with higher scores indicating stronger self‐efficacy [25].

The European Quality of Life 5‐Dimension 5‐Level version score (EQ‐5D‐5L) was used in this study to assess the quality of life and self‐perceived health of the respondents. Five dimensions were assessed: mobility, self‐care, daily activities, pain or discomfort, and anxiety or depression. In the analysis, EQ‐5D‐5L health utility scores were calculated using the Chinese value set; higher scores indicate better health‐related quality of life [26, 27].

The Media Use Scale [28, 29] was used to assess an individual's media consumption behaviors. The scale consists of six items corresponding to six different types of media use behaviors: social communication, self‐presentation, social action, leisure and recreation, access to information through the media, and business transactions. On the Likert scale, each item ranges from 1 to 5. Total scores range from 6 to 30, with higher scores indicating more frequent use of the medium.

The Patient Health Questionnaire (PHQ‐9) was used to assess depressive disorders in the respondents. Each option was rated according to a Likert‐type scale ranging from 0 (not at all) to 3 (almost every day). Scores of 0–4 were defined as no depression, 5–9 as mild depression, 10–14 as moderate depression, 15–19 as moderate to severe depression, and 20–27 as severe depression [30, 31].

The Generalized Anxiety Disorder‐7 (GAD‐7) was used to assess participants' anxiety states. Based on a standardized classification, a score of 0–4 reflects no anxiety, 5–9 reflects mild anxiety, 10–13 reflects moderate anxiety, 14–18 reflects moderate to severe anxiety, and 19–21 reflects severe anxiety [32, 33].

Given the open‐source and freely accessible nature of the aforementioned scales, the research group emailed each author to request authorization. Therefore, we have obtained the necessary permissions to utilize the above scales.

### 2.3 Statistical Analysis

All statistical analyses were conducted using SPSS software for Windows, version 27.0.1 (SPSS Inc). To account for the complex sampling design of this study, one‐way ANOVA was first performed on the collected data to facilitate exploration of the association between each factor and willingness to participate in clinical trials of new drugs. Through the results of the analysis, significant factors were included in subsequent forward stepwise linear regression. Continuous variables were directly included as independent variables in the linear regression, while categorical or hierarchical variables were converted into dummy variables, with one group selected as the reference group to compare willingness to participate in clinical trials of new drugs between subgroups. Age subgroup analysis was performed given that age is closely related to disease burden, risk perception, health information access, and medical decision‐making. Chronic disease status was selected because long‐term disease management, continuing medication use, and perceived treatment needs may alter risk‐benefit assessments of new drug clinical trials. Adjusted *R* ^2^, Akaike information criterion (AIC), and Bayesian information criterion (BIC) were calculated to evaluate model fit. In addition, to assess the accuracy of the analytical model, multicollinearity diagnostics and proportional odds hypothesis score tests were performed. All *p*‐values were from 2‐sided tests, and results were considered statistically significant when *p* < 0.05.

## 3 Results

### 3.1 Sociodemographic Characteristics of the Population

The general characteristics and sociological characteristics of the study population are shown in Table 1. A total of 19,710 participants were included in this study, of whom 9747 (49.50%) were male and 9963 (50.50%) were female. A total of 6672 (33.90%) participants were aged 18–30 years, 8924 (45.30%) participants were aged 31–59 years, and 4114 (20.90%) participants were aged 60 years or older. There was a total of 6497 (33.00%) participants in the eastern region, 4871 (24.70%) in the central region, and 8342 (42.30%) in the western region. 5730 (29.10%) participants had the education level of junior high school and below, 4362 (22.10%) participants had the education of high school and junior college, and 9618 (48.80%) participants had the education level above college. A total of 1590 (8.10%) participants had occupations or professions related to medicine. 13,360 (67.80%) of the participants were married and 6350 (32.20%) were unmarried. 18,408 (93.40%) participants had health insurance (including resident's health insurance, employee's health insurance, new rural cooperative, commercial insurance, and public health care), and 298 (1.50%) participants were with a rare disease or cancer.

**TABLE 1:** Descriptive statistics on the acceptance of participating in clinical trials of new drugs among study participants.

Characteristics	No. of participants (%)	Acceptance scores, mean (SD)	*p* value
Total	19,710 (100.0)	N/A	N/A
Gender			
Male	9747 (49.5)	45.57 (29.48)	<0.001
Female	9963 (50.5)	43.83 (28.58)	
Age (years)			
18–30	6672 (33.9)	41.65 (28.08)	<0.001
31–59	8924 (45.3)	45.97 (29.55)	
≥60	4114 (20.9)	46.85 (29.06)	
Ethnic group			
Han	18,023 (91.4)	44.72 (29.95)	0.614
Minorities (including 38 ethnic groups)	1687 (8.6)	44.35 (30.00)	
Occupation			
Students	4450 (22.6)	40.59 (27.62)	<0.001
Retired	2745 (13.9)	47.48 (29.29)	
Employed	7558 (38.3)	46.00 (29.33)	
No fixed occupation	4957 (25.1)	44.84 (29.32)	
Highest education level			
Junior high school and below	5730 (29.1)	45.56 (29.28)	0.016
High school and junior college	4362 (22.1)	43.96 (28.49)	
College and above	9618 (48.8)	44.51 (29.13)	
Have medical occupations or professions			
No	18,120 (91.9)	44.16 (29.01)	<0.001
Yes	1590 (8.1)	50.69 (28.69)	
Region			
Eastern China	6497 (33.0)	43.84 (29.06)	<0.001
Central China	4871 (24.7)	41.89 (29.19)	
Western China	8342 (42.3)	46.99 (28.76)	
With chronic diseases			
No	14,210 (72.1)	44.87 (29.03)	0.158
Yes	5500 (27.9)	44.22 (29.05)	
With cancer and rare disease			
No	19,412 (98.5)	44.61 (29.04)	0.003
Yes	298 (1.5)	49.71 (28.33)	
Residence in the last 3 months			
Urban	13,697 (69.5)	44.21 (29.11)	<0.001
Rural	6013 (30.5)	45.79 (28.84)	
Marital status			
Unmarried	6350 (32.2)	41.63 (28.10)	<0.001
Married	13,360 (67.8)	46.15 (29.36)	
Have debt			
No	12,298 (62.4)	44.98 (29.13)	0.076
Yes	7412 (37.6)	44.22 (28.88)	
Monthly household income (yuan)			
≤3000	6507 (33.0)	43.59 (28.86)	<0.001
3000–6000	8112 (41.2)	44.73 (28.60)	
>6000	5091 (25.8)	46.05 (29.90)	
Have medical insurance			
No	1302 (6.6)	47.17 (30.90)	0.001
Yes	18,408 (93.4)	44.52 (28.90)	
Supplement taken in the last 24 h			
No	11,491 (58.3)	43.98 (29.28)	<0.001
Yes	8219 (41.7)	45.69 (28.67)	
PHQ‐9			
No depression (0–4)	8322 (42.2)	44.91 (29.91)	<0.001
Mild depression (5–9)	6984 (35.4)	43.44 (27.98)	
Moderate depression (10–14)	2737 (13.9)	44.84 (27.86)	
Moderate to severe depression (15–19)	1253 (6.4)	47.55 (29.12)	
Severe depression (20–27)	414 (2.1)	51.64 (34.09)	
GAD‐7			
No anxiety (0–4)	10,477 (53.2)	44.46 (29.80)	0.008
Mild anxiety (5–9)	6524 (33.1)	44.38 (27.93)	
Moderate anxiety (10–13)	1612 (8.2)	45.82 (26.64)	
Moderate to severe anxiety (14–18)	838 (4.3)	47.84 (29.73)	
Severe anxiety (19–21)	259 (1.3)	44.54 (36.04)	

Abbreviations: GAD‐7, the Generalized Anxiety Disorder‐7; N/A, not applicable; PHQ‐9, the Patient Health Questionnaire; SD, standard deviation.

The mean willingness to participate in clinical trials of new drugs was 44.69 ± 29.04. The mean willingness to participate in clinical trials of new drugs varied by region, with a mean willingness score of 43.84 ± 29.06 in the eastern region, 41.89 ± 29.19 in the central region, and 46.99 ± 28.76 in the western region. The average NGSES score for the surveyed group was 10.79 ± 2.40, the HLS‐SF12 score was 34.22 ± 9.78, the PSSS score was 15.02 ± 3.75, and the media use score was 19.28 ± 5.36.

### 3.2 Factors Associated With Willingness to Participate in Clinical Trials

The results indicate that gender, age, region, occupation, marital status, economic status, health insurance participation, physical and mental health (diagnosis of chronic, rare diseases and cancer, self‐efficacy, perceived stress, and perceived social support), media exposure, and health literacy were significant factors associated with willingness to participate in clinical trials of new drugs (Table 2). The adjusted *R* ^2^, AIC, and BIC of the main model were 0.039, 187967.59, and 188180.59, respectively.

**TABLE 2:** Linear regression analysis of factors associated with the acceptance of participating in clinical trials of new drugs.

	Unstandardized coefficient			
Variable	*B* (95% CI)	SE	Beta (*β*)	*p* value
Constant	18.91 (14.43, 23.40)	2.29	NA	<0.001
Gender (reference: Male)				
Female	−2.29 (–3.10, –1.47)	0.41	−0.04	<0.001
Age group (reference: 18–30 years)				
31–59	3.84 (2.41, 5.27)	0.73	0.07	<0.001
≥60	7.71 (5.79, 9.63)	0.98	0.11	<0.001
Occupation (reference: Students)				
Retired	1.70 (0.18, 3.21)	0.77	0.02	0.028
Employed	1.87 (0.85, 2.88)	0.52	0.03	<0.001
Region (reference: Eastern China)				
Central China	−1.65 (–2.73, –0.56)	0.55	−0.02	0.003
Western China	3.15 (2.20, 4.09)	0.48	0.05	<0.001
Monthly household income (reference: ≤3000)				
3000–6000	0.27 (–0.71, 1.25)	0.50	0.01	0.590
>6000	1.76 (0.60, 2.91)	0.59	0.03	0.003
Medical related occupations (reference: non‐medical related)				
Yes	8.01 (6.52, 9.50)	0.76	0.08	<0.001
With chronic diseases (reference: No)				
Yes	−2.45 (–3.44, –1.46)	0.50	−0.04	<0.001
With cancer and rare disease (reference: No)				
Yes	5.15 (1.85, 8.46)	1.69	0.02	0.002
Place of residence in the last 3 months (reference: Urban)				
Rural	3.00 (2.06, 3.93)	0.48	0.05	<0.001
Marital status (reference: Unmarried)				
Married	2.93 (1.46, 4.40)	0.75	0.05	<0.001
Have medical insurance (reference: No)				
Yes	−3.78 (–5.40, –2.16)	0.83	−0.03	<0.001
Supplements taken in the last 24 h (reference: No)				
Yes	1.44 (0.61, 2.27)	0.43	0.02	0.001
GAD‐7 score [reference: No anxiety (0–4)]				
Mild anxiety (5–9)	0.46 (–0.50, 1.42)	0.49	0.01	0.345
Moderate anxiety (10–13)	1.83 (0.21, 3.44)	0.82	0.02	0.026
Moderate to severe anxiety (14–18)	2.66 (0.54, 4.79)	1.08	0.02	0.014
Severe anxiety (19–21)	−1.94 (–5.62, 1.74)	1.88	−0.01	0.301
PSS‐4 score	0.51 (0.32, 0.70)	0.10	0.04	<0.001
NGSES score	0.34 (0.10, 0.58)	0.12	0.03	0.006
PSSS score	−0.03 (–0.18, 0.12)	0.08	0.00	0.691
Media Use score	0.42 (0.32, 0.52)	0.05	0.08	<0.001
EQ‐5D‐5L score	−0.99 (–4.20, 2.22)	1.64	−0.01	0.546
HLS‐SF12 score	0.21 (0.16, 0.26)	0.03	0.07	<0.001

Abbreviations: CI, confidence interval, SE, standard error.

Compared with males, females (*β* = –2.29 [95% CI: –3.10 to –1.47]) had a lower willingness to participate in clinical trials of new drugs. Residents in rural areas (*β* = 3.00 [95% CI: 2.06 to 3.93]) had a higher willingness to participate in clinical trials of new drugs compared with urban areas. In contrast to the unmarried population, the married population (*β* = 2.93 [95% CI: 1.46 to 4.40]) had a higher willingness to participate in clinical trials of new drugs. Willingness to participate in clinical trials of new drugs was higher among those aged 31–59 years (*β* = 3.84 [95% CI: 2.41 to 5.27]) and 60 years or older (*β* = 7.71 [95% CI: 5.79 to 9.63]) compared with those aged 18–30 years. In addition, compared with the eastern region, the western region (*β* = 3.15 [95% CI: 2.20 to 4.09]) had a higher willingness to participate in clinical trials of new drugs, while the central region (*β* = –1.65 [95% CI: –2.73 to –0.56]) had a lower willingness to participate in clinical trials of new drugs. The retired (*β* = 1.70 [95% CI: 0.18 to 3.21]) and employed (*β* = 1.87 [95% CI: 0.85 to 2.88]) populations had a higher willingness to participate in clinical trials of new drugs than the students. Compared to individuals with non‐medical‐related occupations, those with medical‐related occupations (*β* = 8.01 [95% CI: 6.52 to 9.50]) had a higher willingness to participate in clinical trials of new drugs. Compared to individuals with monthly incomes less than 3000 yuan, those with above 6000 yuan (*β* = 1.76 [95% CI: 0.60 to 2.91]) were more willing to participate in clinical trials of new drugs. In addition, individuals who have taken supplements (*β* = 1.44 [95% CI: 0.61 to 2.27]) have a higher willingness to participate in clinical trials of new drugs.

The results also showed that the NGSES score (*β* = 0.34 [95% CI: 0.10 to 0.58]), PSS‐4 score (*β* = 0.51 [95% CI: 0.32 to 0.70]), media use score (*β* = 0.42 [95% CI: 0.32 to 0.52]), and HLS‐SF12 score (*β* = 0.21 [95% CI: 0.16 to 0.26]) were positively correlated with willingness to participate in clinical trials of new drugs. The PSSS score was not significantly associated with willingness in the main model (*β* = –0.03 [95% CI: –0.18 to 0.12]). People with rare diseases or cancer (*β* = 5.15 [95% CI: 1.85 to 8.46]) were more receptive to clinical trials of new drugs compared to those without. However, people with chronic diseases (*β* = –2.45 [95% CI: –3.44 to –1.46]) had reduced willingness to participate in clinical trials of new drugs compared to people without chronic diseases.

### 3.3 Factors Associated With the Acceptance of Participating in New Drugs in Clinical Trials at Different Ages

Because the regression results for the whole population showed that age was a significant factor in determining willingness to participate in clinical trials of new drugs, we performed a subgroup analysis by age (Table 3). The model fit indices for the age subgroup models were as follows: among participants aged 18–30 years, adjusted *R* ^2^ = 0.014, AIC = 63364.34, and BIC = 63507.26; among participants aged 31–59 years, adjusted *R* ^2^ = 0.033, AIC = 85486.40, and BIC = 85635.42; and among participants aged 60 years or older, adjusted *R* ^2^ = 0.086, AIC = 39046.54, and BIC = 39179.31. In the 18–30 years subgroup, region (western China [*β* = 5.80]) and the two scales (NGSES[*β* = 0.48], and HLS‐SF12 [*β* = 0.14]) were consistent with the overall situation. In the 31–59 years subgroup, similar to the overall population, gender (female [*β* = –2.08]), region (western China [*β* = 3.64]), place of residence in the last 3 months (rural [*β* = 2.78]), economic status (have medical insurance [*β* = –5.58], monthly household income over 6000 yuan [*β* = 2.50]), physical and mental health status (with cancer and rare disease [*β* = 7.41]), having taken supplements within 24 h (*β* = 1.44), and the four scales (PSS‐4 [*β* = 0.51], NGSES [*β* = 0.53], media use [*β* = 0.42], and HLS‐SF12 [*β* = 0.27]) revealed correlations. In the subgroup aged 60 years or older, region (central China [*β* = –9.41]), place of residence in the last 3 months (rural [*β* = 3.23]), physical and mental health status (with chronic diseases [*β* = –6.60]), economic status (have medical insurance [*β* = –12.55], monthly household income over 6000 yuan [*β* = 4.72]), having taken a nutritional supplement within 24 h (*β* = 2.59), and the four scales (PSS‐4 [*β* = 1.08], media use [*β* = 0.49], EQ‐5D‐5L [*β* = 5.93], and HLS‐SF12 [*β* = 0.23]) also showed correlations consistent with the general trend.

**TABLE 3:** Analysis of differences in linear regression model by age subgroup.

	18–30 years		31–59 years		≥60 years	
Variable	*β* (95% CI)	*p* value	*β* (95% CI)	*p* value	*β* (95% CI)	*p* value
Constant	30.73 (23.18, 38.28)	<0.001	22.41 (15.24, 29.58)	<0.001	36.30 (27.31, 45.29)	<0.001
Gender (reference: Male)						
Female	−1.22 (–2.59, 0.15)	0.081	−2.08 (–3.31, –0.85)	<0.001	−1.25 (–2.96, 0.46)	0.151
Region (reference: Eastern China)						
Central China	1.57 (–0.19, 3.33)	0.080	0.35 (–1.26, 1.95)	0.672	−9.41 (–12.05, –6.77)	<0.001
Western China	5.80 (4.22, 7.38)	<0.001	3.64 (2.16, 5.12)	<0.001	−0.69 (–2.65, 1.26)	0.485
With chronic diseases (reference: No)						
Yes	−0.31 (–2.47, 1.85)	0.779	0.13 (–1.29, 1.55)	0.859	−6.60 (–8.42, –4.77)	<0.001
With cancer and rare disease (reference: No)						
Yes	3.68 (–3.00, 10.35)	0.281	7.41 (2.39, 12.44)	<0.001	2.16 (–3.67, 7.98)	0.468
Place of residence in the last 3 months (reference: Urban)						
Rural	1.20 (–0.42, 2.82)	0.147	2.78 (1.33, 4.23)	<0.001	3.23 (1.40, 5.05)	<0.001
Have medical insurance (reference: No)						
Yes	1.86 (–0.38, 4.09)	0.103	−5.58 (–8.55, –2.60)	<0.001	−12.55 (–16.26, –8.84)	<0.001
Monthly household income (reference: ≤3000, yuan)						
3000–6000	−0.99 (–2.70, 0.73)	0.259	0.85 (–0.63, 2.32)	0.261	0.45 (–1.53, 2.43)	0.656
>6000	0.38 (–1.51, 2.26)	0.696	2.50 (0.77, 4.22)	0.005	4.72 (2.04, 7.41)	<0.001
Supplement taken in the last 24 h (reference: No)						
Yes	0.50 (–0.89, 1.88)	0.482	1.44 (0.16, 2.71)	0.027	2.59 (0.80, 4.38)	0.005
GAD‐7 score (reference: No anxiety [0–4])						
Mild anxiety (5–9)	−0.90 (–2.52, 0.71)	0.272	0.67 (–0.78, 2.12)	0.364	1.57 (–0.55, 3.70)	0.146
Moderate anxiety (10–13)	1.88 (–0.73, 4.49)	0.159	1.51 (–1.02, 4.04)	0.243	0.63 (–2.83, 4.09)	0.722
Moderate to severe anxiety (14–18)	2.64 (–0.55, 5.83)	0.104	2.21 (–1.24, 5.65)	0.210	3.50 (–1.60, 8.60)	0.178
Severe anxiety (19–21)	−3.64 (–8.68, 1.39)	0.156	0.02 (–5.92, 6.02)	0.986	2.70 (–11.26, 16.67)	0.704
PSS‐4 score	0.02 (–0.31, 0.35)	0.906	0.51 (0.22, 0.79)	<0.001	1.08 (0.66, 1.49)	<0.001
PSSS score	−0.09 (–0.35, 0.16)	0.481	0.16 (–0.07, 0.38)	0.176	−0.10 (–0.45, 0.25)	0.574
NGSES score	0.48 (0.07, 0.89)	0.021	0.53 (0.17, 0.89)	<0.001	−0.07 (–0.60, 0.46)	0.799
Media Use score	0.09 (–0.10, 0.28)	0.353	0.42 (0.27, 0.57)	<0.001	0.49 (0.31, 0.66)	<0.001
EQ‐5D‐5L score	−4.23 (–9.82, 1.37)	0.139	−3.70 (–8.97, 1.57)	0.169	5.93 (0.12, 11.75)	0.046
HLS‐SF12 score	0.14 (0.06, 0.23)	0.001	0.27 (0.20, 0.35)	<0.001	0.23 (0.12, 0.33)	<0.001

Abbreviations: CI, confidence interval.

### 3.4 Factors Associated With Willingness to Participate in Clinical Trials Among Patients With Chronic Diseases

Chronic diseases were the main focus of clinical trials of new drugs, so we grouped these patients to explore the influencing factors that affect patients' willingness to participate in clinical trials of new drugs (Table 4). The model fit indices were 0.046 for adjusted *R* ^2^, 135411.90 for AIC, and 135600.94 for BIC in the subgroup without chronic diseases, and 0.036 for adjusted *R* ^2^, 52489.05 for AIC, and 52654.37 for BIC in the subgroup with chronic diseases. In the subgroup without chronic diseases, similar to the overall population, gender (female [*β* = –2.21]), age (31–59 years [*β* = 3.25], 60 years or older [*β* = 11.55]), region (western China [*β* = 4.51]), occupation (employed [*β* = 2.70]), marital status (married [*β* = 2.46]), place of residence in the last 3 months (rural [*β* = 2.32]), having medical occupations or professions (*β* = 8.28), economic status (have medical insurance [*β* = –3.55], monthly household income over 6000 yuan [*β* = 1.77]), having taken supplements within 24 h (*β* = 1.14), and four scales (PSS‐4 [*β* = 0.55], NGSES [*β* = 0.33], media use [*β* = 0.39], and HLS‐SF12 [*β* = 0.18]) also revealed consistent results.

**TABLE 4:** Analysis of differences in linear regression models between subgroups with or without chronic diseases.

Variable	Without chronic diseases		With chronic diseases	
	*B* (95% CI)	*p* value	*B* (95% CI)	*p* value
Constant	18.17 (12.64, 23.71)	<0.001	23.51 (15.18, 31.85)	<0.001
Gender (reference: Male)				
Female	−2.21 (–3.16, –1.25)	<0.001	−2.49 (–4.03, –0.94)	0.002
Age group (reference: 18–30, years)				
31–59	3.25 (1.70, 4.80)	<0.001	5.35 (1.69, 9.00)	0.004
≥60	11.55 (9.22, 13.88)	<0.001	3.48 (–0.61, 7.58)	0.100
Region (reference: Eastern China)				
Central China	−0.95 (–2.21, 0.31)	0.138	−3.17 (–5.28, –1.06)	0.003
Western China	4.51 (3.39, 5.62)	<0.001	−0.45 (–2.24, 1.34)	0.620
Occupation (reference: Students)				
Retired	0.90 (–1.25, 3.05)	0.410	2.01 (–0.19, 4.21)	0.070
Employed	2.70 (1.55, 3.85)	<0.001	−1.10 (–3.28, 1.08)	0.320
Have medicine occupations or professions (reference: No)				
Yes	8.28 (6.60, 9.96)	<0.001	6.80 (3.54, 10.06)	<0.001
Place of residence in the last 3 months (reference: Urban)				
Rural	2.32 (1.22, 3.42)	<0.001	4.24 (2.46, 6.02)	<0.001
Marital status (reference: Unmarried)				
Married	2.46 (0.85, 4.08)	0.003	3.40 (–0.17, 6.98)	0.060
Have medical insurance (reference: No)				
Yes	−3.55 (–5.33, –1.77)	<0.001	−4.75 (–8.70, –0.80)	0.020
Supplement taken in the last 24 h (reference: No)				
Yes	1.14 (0.16, 2.12)	0.023	2.12 (0.55, 3.69)	0.008
Monthly household income (reference: ≤3000)				
3000–6000	0.63 (–0.53, 1.78)	0.148	−0.92 (–2.78, 0.94)	0.331
>6000	1.77 (0.43, 3.10)	0.010	1.31 (–0.99, 3.61)	0.270
GAD‐7 score [reference: No anxiety (0–4)]				
Mild anxiety (5–9)	−0.22 (–1.35, 0.91)	0.699	2.27 (0.44, 4.10)	0.020
Moderate anxiety (10–13)	1.03 (–0.90, 2.96)	0.296	3.67 (0.76, 6.63)	0.010
Moderate to severe anxiety (14–18)	0.68 (–1.85, 3.21)	0.598	7.23 (3.32, 11.14)	<0.001
Severe anxiety (19–21)	−5.49 (–10.08, –0.90)	0.019	4.77 (–1.44, 10.99)	0.130
PSS‐4 score	0.55 (0.33, 0.77)	<0.001	0.36 (–0.01, 0.73)	0.060
PSSS score	0.10 (–0.08, 0.28)	0.263	−0.30 (–0.58, –0.02)	0.037
NGSES score	0.33 (0.04, 0.62)	0.025	0.33 (–0.11, 0.77)	0.140
Media Use score	0.39 (0.28, 0.51)	<0.001	0.41 (0.23, 0.58)	<0.001
EQ‐5D‐5L score	−1.66 (–5.97, 2.64)	0.448	−1.43 (–6.28, 3.42)	0.560
HLS‐SF12 score	0.18 (0.12, 0.24)	<0.001	0.27 (0.17, 0.36)	<0.001
No. observations	14,210		5500	
Adjusted *R* ^2^	0.046		0.036	
AIC	135411.90		52489.05	
BIC	135600.94		52654.37	

Abbreviations: AIC, Akaike information criterion; BIC, Bayesian information criterion; CI, confidence interval.

In the subgroup with chronic diseases, gender (female [*β* = –2.49]), age (31–59 years [*β* = 5.35]), region (central China [*β* = –3.17]), place of residence in the last 3 months (rural [*β* = 4.24]), having medical occupations or professions (*β* = 6.80), economic status (have medical insurance [*β* = –4.75]), physical and mental health status (moderate anxiety [*β* = 3.70], moderate to severe anxiety [*β* = 7.23]), having taken supplements within 24 h (*β* = 2.12), and three scales (PSSS score [*β* = –0.30], media use [*β* = 0.41], and HLS‐SF12 [*β* = 0.27]) also showed correlations consistent with the general trend.

## 4 Discussion

This national, cross‐sectional study in China revealed that people have a low willingness to participate in clinical trials of new drugs, with a mean score of 44.69 out of 100. Gender, age, marital status, occupation, area of residence, income, health insurance, physical and mental health status (chronic diseases, rare diseases or cancer, anxiety, perceived stress, self‐efficacy), media use, and health literacy significantly influence willingness. Subgroup analysis demonstrated consistent trends across age and chronic disease subgroups. These findings add multicenter, general‐population evidence to existing Chinese studies, which have mainly focused on public awareness of drug clinical trials in specific regions [18] or on healthy volunteers participating in clinical trials [19].

The public's willingness to participate in clinical trials of new drugs was relatively low in China (44.69 ± 29.04 out of 100) compared with other countries. A cross‐sectional study conducted in the United States [34] demonstrated that 90% of participants believed in the safety of clinical trials and 74.5% of participants were willing to take part in clinical trials. Two studies conducted in Saudi Arabia in 2016 [11] and 2018 [14] noted willingness to participate in clinical trials with mean scores of 61.5 ± 28.0 and 70.1 ± 16.4 out of 100, respectively. A Chinese online survey in northern inland cities reported that 52.9% of respondents were willing to participate in drug clinical trials [18], while a Wuhan‐based study of healthy volunteers emphasized the need for greater social/family support and more positive media reports about clinical trial participation [19]. Although differences in study population, measurement methods, and social context limit direct comparison, these results collectively suggest that willingness to participate in clinical trials of new drugs remains suboptimal and requires targeted public communication. The unwillingness to engage in clinical trials may stem from structural barriers (e.g., requirement of extra time and efforts including tests, costs, and travel), procedural barriers (e.g., lack of public information and lack of public awareness), cognitive barriers (e.g., apprehension regarding the potential adverse effects of new drugs and concerns about interfering with established treatment methods), and psychological barriers (e.g., distrust of medical research and reluctance to serve as a test subject) [35]. Another possible reason may be that relatively low drug expenses [36] and high reimbursement rates [37, 38] for medical insurance discourage people from seeking new treatment options in China.

Age is shown to be an important factor influencing willingness to participate in clinical trials of new drugs. Our study indicates that individuals aged 31–59 demonstrate a greater willingness to engage in such trials, as compared to those aged 18–30. According to a cross‐sectional study of cancer patients conducted in United States [9], individuals aged 18–24 displayed a lower inclination to participate in clinical trials than those aged 55–64, which is aligned with our study. Our study further identified that individuals aged above 60 also have a higher willingness to participate in clinical trials of new drugs, which might be related to multiple comorbid conditions and subsequent high demand for medications in the elderly. Our study also points to gender as an important influential factor, in which males have a higher willingness to participate in clinical trials of new drugs compared to females. A previous study also states that more quantities of males participate in clinical trials than females [8]. Possible reasons are that most females have reproductive plans and are more concerned about the potential adverse impact of examined new drugs on their offspring. In addition, reduced willingness was seen in unmarried people in our study. However, a previous study in Saudi Arabia [14] showed that single people (mean score, 71.4 ± 16.0) had a more positive attitude toward participation in clinical trials compared to married people (mean score, 68.4 ± 16.8; *p* = 0.021). Similarly, another study in the United States [15] revealed that single people have a higher willingness to participate in clinical trials of new drugs compared to married people (adjusted odds ratio, 1.68; 95% CI: 1.04 to 2.73). The inconsistency between previous studies and our study is possibly due to geographical and cultural differences and needs further investigation.

Health literacy, as an indicator of patients' ability to obtain, process, and understand basic health information and make appropriate health decisions [39], was found to be positively correlated with the willingness to participate in clinical trials of new drugs. People with poor health literacy were more passive in seeking health information [40], which results in lacking knowledge of clinical trials. Meanwhile, low‐income populations are at confirmed risk of poor health literacy [41, 42], which may account for the reduced willingness observed in our study. Moreover, people with low incomes are more concerned about who pays for clinical trials [12], possibly discouraging their participation decisions. However, the absence of dependable sources of information [43] and substantial social support [44] can similarly render low‐income populations particularly vulnerable to uncertainty, thereby diminishing their propensity to engage in clinical trials. Furthermore, cultural perceptions have been shown to influence these outcomes. In areas or communities where low‐income groups are concentrated, misconceptions and prejudices about medical research may be prevalent, leading to the belief that clinical trials of new drugs are unsafe [34]. Such deeply entrenched cultural perceptions are prevalent among low‐income groups, further exacerbating their rejection of new drug clinical trials. Similar to previous studies [6], our findings indicate that rural populations have a higher acceptance of participating in clinical trials of new drugs compared to urban populations. Western China had the highest willingness to participate in clinical trials of new drugs, which may be due to the relatively insufficient medical resources in western China.

Interestingly, reduced willingness was seen in patients with chronic diseases, while increased willingness was seen in patients with cancer. Patients with chronic diseases usually have well‐established treatment regimens and a wider choice of drugs with exact efficacy, which possibly reduces their demand for new therapy. In contrast, cancer patients have limited therapy regimens and poor prognosis. They are physically weaker and are more urgent to seek new treatment options, which may explain the higher willingness in these patients.

Our study also found that pressure and anxiety significantly increase the willingness of people to participate in clinical trials of new drugs. Pressure [45] and anxiety [46] affect the decision‐making process. People in high‐pressure situations are more likely to make high‐risk decisions [45]. Anxious people are reassured by searching the Internet for abundant health information [47], which may increase their knowledge of clinical trials of new drugs. This explains why people with higher PSS‐4 scores or GAD‐7 scores tend to participate in clinical trials of new drugs. The willingness to participate in a clinical trial is also influenced by an individual's perceived self‐efficacy beliefs. There is a strong mediating effect between self‐efficacy and decisional conflict about accepting participation in clinical trials of new drugs [48]. As self‐efficacy increases, it reduces decisional conflict, which in turn may enhance individuals' receptiveness to enrolling in clinical trials of new drugs. A previous study showed similar results by using a different self‐efficacy measure scale, which further validated the relationship between self‐efficacy and willingness to participate in clinical trials [49]. As shown in a national communication campaign to raise public awareness of clinical trials in the United States [17], increased self‐efficacy is a crucial behavioral determinant that makes it easier to consider participation in clinical trials. Policy measures should focus on promoting self‐efficacy among individuals who have lower confidence in their ability to participate in clinical trials. Measures can involve the development of targeted educational programs that provide information on the benefits and importance of clinical research, highlighting success stories of trial participants, and addressing common misconceptions and fears.

Of note, our study indicates a clear correlation between media use and the willingness to embrace new drug trials. The media plays a pivotal role in disseminating information about these clinical trials to the general public. The study by Sang [10] showed that an impressive 86% of the participants became aware of clinical trials through media channels. Another study by Leiter [16] indicated that people utilizing the internet displayed higher levels of clinical trial awareness than those who did not (OR, 2.13; 95% CI: 1.52 to 3.00). Multimedia campaigns could be an effective strategy to enhance public awareness, including sharing successful participant experiences and advocating individual and social benefits of clinical trials of new drugs.

To further explore the robustness and consistency of the main research findings, we conducted subgroup analyses by age and chronic disease status. These two subgroup variables were selected a priori because they are directly relevant to clinical trial recruitment and participation decisions. Age may reflect differences in disease burden, risk perception, treatment needs, and autonomy in medical decision‐making. Chronic disease status may reflect differences in established medication use, perceived stability of current treatment, and expected benefit from investigational drugs. The age‐subgroup results suggest that economic factors, media use, and chronic diseases played an inconsistent role among people aged 18–30 years, 31–59 years, and 60 years or older. The subgroup analysis by chronic disease status revealed that anxiety, which is susceptible to chronic diseases [50], had an inconsistent impact on willingness. In other words, the phenomenon that willingness to participate in clinical trials of new drugs increased along with worsening anxiety was only demonstrated in the group with chronic diseases.

Multiple factors (e.g., income, health literacy, media exposure, and anxiety) jointly point out that understanding of clinical trials is a cornerstone to successful participation in clinical trials. A study in 2020 found that up to 36% of respondents admitted they understood clinical research not at all or not very well [51]. It demonstrated that people do not want to be involved in clinical trials of new drugs partly due to inadequate access to information about clinical trials. Therefore, measures should focus on establishing the correct perception and enhancing health communication of clinical trials of new drugs. Policies should aim to bridge this gap by implementing awareness campaigns through alternative channels such as community centers, healthcare facilities, dedicated websites, helplines, and community‐based outreach programs.

Clinical trials of new drugs serve as a critical bridge between preclinical research and clinical translation, extending far beyond scientific validation to play an indispensable role in advancing novel drug development and clinical innovation. Given China's large population, there is a substantial opportunity to recruit diverse participants for new drug clinical research. Investigating public attitudes toward such trials, particularly the reasons underlying refusal to participate, can help identify and engage potential candidates, thereby expanding the pool of eligible subjects. Previous Chinese studies have provided valuable evidence on drug clinical trial awareness and willingness in northern inland cities [18], healthy volunteers in Wuhan [19], and patients with cancer or other specific diseases [7]. Our study complements this domestic evidence by focusing on the general population across multiple provinces and by identifying cross‐regional similarities and differences in influencing factors.

Several significant limitations inherent in this study must be acknowledged. Firstly, reliance on a questionnaire incorporating self‐reported and self‐assessed data may have introduced potential biases into the outcomes. The self‐reported willingness to participate may differ to some extent from the actual willingness to engage in a new drug clinical trial. Secondly, the geographic representation was uneven. While the study encompassed participants from multiple Chinese cities, it did not cover all urban areas nationwide. Since non‐respondents did not return the questionnaire, the demographic characteristics of non‐respondents in this study could not be accounted for or analyzed. However, a strict quota sampling method was adopted to align with the 2021 China Population Census, thereby ensuring the representativeness of the respondent population. Moreover, the research was confined to community settings, thereby excluding hospitalized patients from the analytical datasets, potentially restricting the generalizability of the findings. Thirdly, the study employed a cross‐sectional design, which inherently restricts the ability to infer causality among variables. Fourthly, this survey assessed willingness to participate in clinical trials of new drugs in general and did not collect information on specific investigational drug types, therapeutic categories, disease indications, trial phases, or expected risk‐benefit profiles. These factors may influence participation decisions and should be incorporated into future research and trial‐recruitment strategy studies. The adjusted *R* ^2^ values were relatively low, indicating that the included variables explained only a modest proportion of variation in willingness. The regression models should be interpreted as association models rather than prediction models. Therefore, to enhance our comprehension of the determinants influencing willingness to participate in clinical trials of new drugs, future research should concentrate on elucidating causal linkages through longitudinal or experimental designs and on distinguishing willingness across therapeutic categories.

In conclusion, this national, cross‐sectional study in China revealed low public willingness to participate in clinical trials of new drugs. The willingness was affected by marital status, occupation, area of residence, income, health insurance, physical and mental health status (chronic diseases, rare diseases or cancer, anxiety, perceived stress, self‐efficacy), media use, and health literacy. Multiple measures are needed to bridge the knowledge gap and enhance health communication to raise public awareness of clinical trials of new drugs.

## Funding

This research was partially supported by the National Natural Science Foundation of China (72304009) and SIRG—CityU Strategic Interdisciplinary Research Grant (7020093).

## Conflicts of Interest

All authors declare no conflicts of interest.

## Data Availability

The PBICR project has been ongoing from 2020 to the present, with an annual multi‐center extensive sample size transect survey. We usually distinguish between different years of surveys by the suffix (e.g., PBICR2022 denotes surveys conducted by PBICR in 2022). The historical materials and any associated protocols supporting this study's findings are available from the corresponding authors upon request (email: bjmuwuyibo@outlook.com).
